# Superoxide Dismutase Mimic, MnTE-2-PyP Enhances Rectal Anastomotic Strength in Rats after Preoperative Chemoradiotherapy

**DOI:** 10.1155/2020/3509859

**Published:** 2020-04-13

**Authors:** Yu Yang, Qi Wang, Jiajun Luo, Yue Jiang, Rui Zhou, Shilun Tong, Zhihua Wang, Qiang Tong

**Affiliations:** ^1^Department of Gastrointestinal Surgery I Section, Renmin Hospital of Wuhan University, Wuhan 430060, China; ^2^Hubei Province Key Laboratory of Allergy and Immunology, School of Basic Medical Sciences, Wuhan University, Wuhan 430071, China; ^3^Central Laboratory, Renmin Hospital of Wuhan University, Wuhan 430060, China

## Abstract

**Background:**

Rectal cancer is one of the malignant diseases with high morbidity and mortality in the world. Currently, surgical resection is the main treatment method, and preoperative chemoradiotherapy (CRT) is widely used in clinical application to increase resectability and decrease the local recurrence rate. However, CRT increases the risk of colon anastomotic leak, and currently, there are no FDA approved treatments against this side effect. It is essential to develop new drugs to reduce postoperative anastomotic leak after preoperative CRT.

**Methods:**

90 rats underwent standard resection and intestine anastomosis treatment and were divided into six groups for different treatments. During the relaparotomy, bursting pressure of anastomosis was measured and intestinal segments were taken for histopathologic examination and biochemical analyses. RT-PCR and ELISA were applied to measure matrix metalloproteinase (MMP) mRNA and protein levels. Blood vessels were observed by immunohistochemistry, and collagen deposition was observed by Picrosirius Red staining.

**Results:**

Preoperative CRT reduced the postoperative anastomotic strength. MnTE-2-PyP increased the bursting pressure and hydroxyproline levels of intestine anastomosis after CRT treatment. Mechanically, MnTE-2-PyP decreased the MMP levels and increased microvessel density (MVD) and collagen deposition. The MMP inhibitor doxycycline had a positive effect on anastomosis healing, but was inferior to MnTE-2-PyP.

**Conclusions:**

MnTE-2-PyP enhanced intestine anastomotic strength in rats with preoperative CRT. Specifically, MnTE-2-PyP decreased MMP levels and increased MVD in anastomosis. Therefore, MnTE-2-PyP may be helpful in the prevention of anastomotic leak after preoperative CRT.

## 1. Introduction

Colorectal cancer is the third most common malignancy and the fourth leading cause of cancer morbidity and mortality in the world, which threatens human health seriously [1]. One third of patients diagnosed with colorectal cancer have a tumor located in the rectum. Although surgery is the main treatment for locally advanced rectal cancer (LARC), the effect of surgery alone was not satisfactory. There are increasing modified strategies for the treatment of rectal cancer; preoperative chemoradiotherapy (CRT) has been gaining acceptance as a therapeutic method in clinical trials. It has been reported that preoperative CRT improves local control, sphincter preservation, and toxicity when compared to postoperative CRT [2]. More and more studies have certified that preoperative CRT can shrink the primary tumor of rectal cancer, reduce the tumor stage, improve the radical resection rate, increase the rate of anus preservation, and decrease local recurrence [3]. Therefore, preoperative CRT has been recommended by the National Comprehensive Cancer Network (NCCN) as the standard treatment method for patients with LARC [4, 5].

However, preoperative CRT has been reported to increase postoperative complications especially the incidence of clinical anastomotic leak (AL) independently in rectal cancer [6, 7]. The reason why there are more AL after CRT is inconclusive. It has been reported that radiation can generate free radicals or reactive oxygen species (ROS), leading to oxidative stress [8]. The ROS generated by ionizing radiation and excessive oxidative stress can initiate DNA and non-DNA damage, oxidize lipids and proteins, and even cause tissue damage [9, 10]. Additionally, the generation of ROS and their by-products was considered to mediate the cytotoxic effects of chemotherapy, which may lead to cell damage [11]. During preoperative CRT, the combination of radiation and chemotherapy drugs resulted in generation of redundant ROS and excessive oxidative stress and caused DNA damage and even tissue damage, which may play important roles in the increasing AL after CRT in rectal cancer patients.

Superoxide dismutase (SOD) mimic, a synthetic compound, can simulate the function of native superoxide dismutase and scavenge ROS effectively. Cationic manganese- (Mn-) substituted N-pyridylporphyrins (MnPs) were initially developed as powerful SOD mimic, and MnPs, which regulate intracellular redox environment, have reached the stage of clinical application [12]. It has been reported that intraperitoneal injection of MnTE-2-PyP (BMX-010, AEOL10113) during radiotherapy performed on the lower abdomen of rats significantly reduced radiation damage to normal tissues such as the skin, prostate, and testis [13], while a similar compound, MnTnBuOE-2-PyP (BMX-001), protected normal tissue effectively while enhancing radiotherapy and chemotherapy treatment in colorectal cancer [14]. It is noteworthy that MnPs have been reported to improve epidermal wound healing. Bellot et al. demonstrated that MnTE-2-PyP treatment can promote wound closure by accelerating neotissue formation compared to the nontreated group in the rodent model [15]. Likewise, Luo et al. have also shown that MnPs improve the poor wound healing in a streptozotocin-induced type I diabetes rat model [16]. However, whether MnPs can promote anastomotic healing and reduce the occurrence of AL after preoperative CRT has yet not been reported.

Matrix metalloproteinases (MMPs) are a group of endopeptidases which have great homology in structure and can degrade almost all extracellular matrix (ECM) proteins. At least 24 different MMPs have been found in human, which are further subdivided according to structure and substrate specificity [17]. MMP-1, MMP-8, MMP-13, and MMP-18 are called collagenases, which play important roles in the degradation of mesenchymal ECM because of ability to cleave type I, II, III, and X collagen. Stromelysins, MMP-3 and MMP-10, have a wide range of substrates such as collagens, gelatins, fibronectin, laminin, and elastin. MMP-12, MMP-19, and MMP-20 are hetypal MMPs, but they cannot be classified as collagenases or stromelysins. MMP-2 and MMP-9 are also named gelatinases and digest denatured collagen and type IV collagen, which are associated with cell invasion. MMP-11, MMP-21, and MMP-28, furin-activatable MMPs, can be activated intracellularly by furin-like proteases [18]. It has been found that selective MMP inhibition can increase anastomotic bursting pressure during the healing process [19, 20], which suggested that MMP may be involved in the healing of intestinal wounds. In addition, oxidative stress is related to the expression and activity of MMPs [21, 22].

In the present study, we established standard resection and colon anastomosis model in rats (because it was difficult to anatomically distinguish colon and rectum in rats), and we found that MnTE-2-PyP promoted anastomotic strength and decreased incidence of intestine AL after preoperative CRT in rats. MnTE-2-PyP effectively accelerated anastomotic healing through inhibiting MMPs, increasing microvessel density and collagen deposition in rat colon tissues. As a broad-spectrum MMP inhibitor, doxycycline was also studied, and its effect on anastomotic healing was compared with MnTE-2-PyP. The results of this study laid a theoretical foundation for clinical application of MnTE-2-PyP on reducing the incidence of postoperative AL in rectal cancer patients after preoperative CRT.

## 2. Materials and Methods

### 2.1. Animals

The present study involved 90 adult male Wistar rats (8–10 weeks), weighing 200–250 g, which were purchased from the Experimental Animal Center of Wuhan University (Wuhan, China). Prior to surgery, rats received free access to food and water in a 12 h light/dark room with a constant temperature (22 ± 2°C) and humidity (40–70%). All experiments were approved by the Animal Experimental Ethics Committee of Wuhan University (Wuhan, China).

### 2.2. Surgical Procedures

All rats were weighed after 12 h of preoperative fasting and anesthetized by intraperitoneal injection of 2% phenobarbital sodium solution (60 mg/kg). After anesthesia, the four limbs of the rats were fixed and the abdomen coat was shaved. Rats can breathe spontaneously during the surgery. A heat lamp was used to keep body temperature at about 37°C. The abdominal cavity was entered through a median incision about 2 cm in the lower abdomen, and then, 1 cm of the left colon was resected. The fecal content at the anastomotic ends was removed. Standardized single-layer end-to-end anastomosis was performed. All surgeries were performed by the same surgeon. All rats were given drinking water after surgery and small amount of food after 12 h.

### 2.3. Treatment Groups and Experimental Design

The rats were randomly divided into 6 groups as follows:

1. Group 1 (control group): standard resection and colon anastomosis treatment (abbreviated as “operation”)
2. Group 2 (sham group): (CRT simulation) plus (operation) plus (PBS)
3. Group 3 (sham+MnTE-2-PyP group): (CRT simulation) plus (operation) plus (MnTE-2-PyP treatment)
4. Group 4 (CRT group): (CRT) plus (operation) plus (PBS)
5. Group 5 (CRT+MnTE-2-PyP group): (CRT) plus (operation) plus (MnTE-2-PyP treatment)
6. Group 6 (CRT+doxycycline group): (CRT) plus (operation) plus (doxycycline treatment)

**Figure 1:**
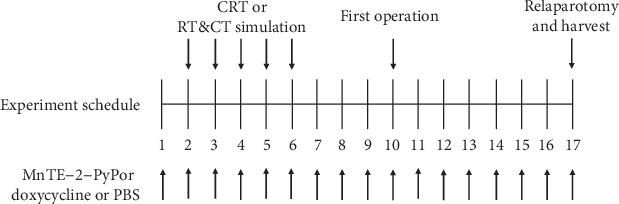
Diagram of experimental design. All rats underwent standard left colon resection and single-layer end-to-end anastomosis of the colon. Relaparotomy was performed after 7 days. CRT or CRT simulation was applied before the first operation as planned. Rats were injected with MnTE-2-PyP or doxycycline or PBS throughout the study as indicated. There were 15 rats in each group.

In brief, there were 15 rats in each group. These rats were weighed at the beginning of the study, before the first operation, and before relaparotomy. All of the groups underwent standard left colon resection and single-layer end-to-end anastomosis of the colon. In two sham groups (groups 2 and 3), chemotherapy (CT) and radiotherapy (RT) simulation was performed before anastomosis, group 2 received phosphate-buffered saline (PBS, 5 mg/kg per day, intraperitoneally), and group 3 received additional MnTE-2-PyP (5 mg/kg per day, intraperitoneally) from the day before CRT simulation was applied until all rats were sacrificed. In CRT groups (groups 4, 5, and 6), CT plus RT was performed before anastomosis, group 4 received PBS, group 5 received additional MnTE-2-PyP, and group 6 received additional doxycycline (15 mg/kg per day, intraperitoneally) from the day before CRT was applied until sacrificed. On postoperative day 7, all groups of rats underwent relaparotomy and bursting pressure (BP) was measured. After measurement of BP, all rats were sacrificed and a 2 cm colon segment containing the anastomotic area was taken for histopathologic examination and biochemical analyses. The experimental design is shown in Figure 1.

### 2.4. CRT Application

Radiotherapy, under anesthesia, began 8 days before the anastomosis. A total of 20 Gy RT was performed on the lower abdomen at a dose of 4 Gy/day for 5 days. Irradiation was performed with 6 MeV of X-rays by a linear accelerator (X-RAD 225; Varian Medical Systems, Inc.). Eight days before the anastomosis, 5-fluorouracil was intraperitoneally injected at a dose of 10 mg/kg for 5 days. The dose range of RT and 5-fluorouracil was explored by referring Oberley-Deegan et al. [13] and Biert et al. [23], respectively. And then, the exact dose of RT and 5-fluorouracil was decided based on the preliminary experiment.

### 2.5. CRT Simulation

Anesthesia was applied daily, but RT was not given. Surgical procedures were applied at the same time to the groups receiving RT. Eight days before the anastomosis, intraperitoneal saline was injected for 5 days at the same volume as CT. Surgical procedures were applied at the same time to the groups receiving CT.

### 2.6. Quantitative Real-Time PCR (qRT-PCR)

The total RNA was extracted from rat tissues by using TRIzol Reagent (Thermo Fisher, USA). Then, RNA was reverse transcribed to cDNA using reverse transcriptase and Oligo dT primers (Takara, Japan). The cDNA was then amplified with specific primers by PCR. The primers used for PCR were listed as follows: MMP-2: forward 5′-GTC GCC CAT CAT CAA GTT CC-3′, reverse 5′-GCA TGG TCT CGA TGG TGT TC-3′; MMP-3: forward 5′-ATG ACA GGG AAG CTG GAC TC-3′, reverse 5′-CTG GAG AAT GTG AGT GGG GT-3′; MMP-8: forward 5′-TGA TCC TGG TGC CTT GAT GT-3′, reverse 5′-TGT GGT GGC AGC ATC AAA TC-3′; MMP-9: forward 5′-AGG ATG GTC TAC TGG CAC AC-3′, reverse 5′-GTG CAG GAC AAA TAG GAG CG-3′; MMP-12: forward 5′-CAC TCC AGA CAT GAA GCG TG-3′, reverse 5′-GAA TAC CGG GCC CAG GAT AA-3′; MMP-13: forward 5′-CCT AAG CAC CCC AAA ACA CC-3′, reverse 5′-GGG AAG TTC TGG CCA AAA GG-3′; and 18S rRNA: forward 5′-CGG CTA CAT CCA AGG AA-3′, reverse 5′-GCT GGA ATT ACC GCG GCT-3′. The conditions for PCR were as follows: 95°C for 3 min, followed by 40 cycles of 10 s at 95°C, 10 s at 60°C, and 15 s at 70°C, followed by heating from 65°C to 95°C.

### 2.7. Measurement of Bursting Pressure

Bursting pressure was measured *in vivo*. Intraperitoneal adhesion was separated, and the colon at the anastomosis was dissociated. The colon was ligated with silk thread and severed at the distal 5 cm of the anastomosis. The pressure gauge was connected to the colon at one end and the syringe pump at the other end. Normal saline was injected at a rate of 2 ml/min until the anastomosis burst. The maximum pressure shown by the pressure gauge was recorded as bursting pressure value.

### 2.8. Measurement of Hydroxyproline

The hydroxyproline assay kit was used according to the manufacturer's instructions (Nanjing Jiancheng Bioengineering Institute, China). Hydroxyproline levels of colon anastomosis were tested following the manufacturer's instructions.

### 2.9. Enzyme-Linked Immunosorbent Assay (ELISA)

Rat MMP-2 ELISA kit was purchased from Elabscience Biotechnology Co., Ltd. (Wuhan, China); rat MMP-9 ELISA kit was purchased from Yaji Biological (Shanghai, China). MMP-2 and MMP-9 levels of colon anastomosis were tested following the manufacturer's instructions.

### 2.10. Picrosirius Red Staining

The Picrosirius Red Stain Kit was purchased from Qianchen Biotechnology Co., Ltd. (Shanghai, China). Collagen components in anastomotic tissues were observed under a microscope. Three independent observers assessed and scored the existence of collagen deposition in the anastomotic tissue. The scoring criteria were as follows: 0 no collagen deposition, 1 occasional collagen deposition, 2 slight collagen deposition, 3 abundant collagen deposition, and 4 confluent fibers. All observers were blinded to experimental groups.

### 2.11. Immunohistochemistry

Paraffin-embedded tissue samples were cut into 4 *μ*m thick sections and mounted on poly-L-lysine coated slides. Anti-rat CD34 antigen monoclonal antibody was purchased from Zhongshan Goldenbridge Biotechnology Co., Ltd. (Beijing, China), and primary antibody binding was visualized using a DAKO EnVision kit (DAKO, Glostrup, Denmark) following the manufacturer's instructions.

### 2.12. Microvessel Density Evaluation

Microvessel was measured by the average number of CD34-positive vessels. We choose five most representative hot spots to observe and use a 400x magnification field to count the number of microvessels. Determined microvessel density (MVD) was expressed as the number of stained microvessels per optical field. The vessel was composed by any CD34-positive cell or cell cluster, and the diameter less than eight red blood cells was counted as a microvessel, as described in the Weidner method. When microvessel counts differed by more than 10 per high power field (HPF), samples would be counted again a week later until recording differences were below 10 counts. The mean number of microvessel per HPF across five hot spots for every sample was defined as the MVD.

### 2.13. Statistical Analysis

GraphPad Prism version 7 was used for statistical analysis, and all values were presented as means ± SD. ANOVA was used for measurement data. Student's *t*-test was used for pairwise comparisons. Statistical significance was defined as *P* < 0.05.

## 3. Results

### 3.1. General Evaluation

**Figure 2:**
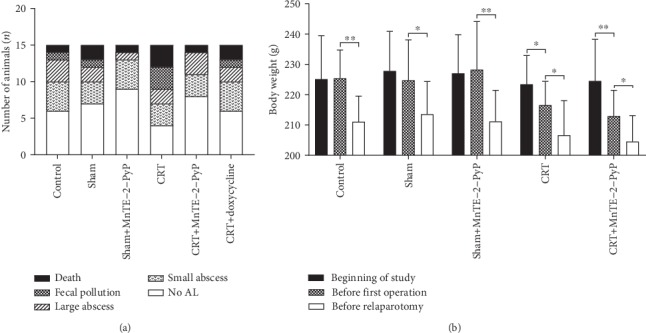
General evaluation of rats in each group. (a) The number of fecal peritonitis death and AL occurred in all experimental groups. (b) Body weight change of rats in the first five groups; rats were weighed at the beginning of the study, during the first operation, and during relaparotomy. Unpaired Student's *t*-test was used to calculate statistical significance. Results are presented as means ± SD. ^∗^*P* < 0.05, ^∗∗^*P* < 0.01.

The AL model in rats was established according to experimental requirements, and macroscopic parameters were used to analyze and verify. Ten rats died after the first operation due to diffuse peritonitis (one in the control group, two in the sham group, one in the sham+MnTE-2-PyP group, three in the CRT group, one in the CRT+MnTE-2-PyP group, and two in the CRT+doxycycline group). The types of AL and mortality in groups 1 to 6 are summarized in Figure 2(a). Compared with the CRT group, the number of AL in the CRT+MnTE-2-PyP group or the CRT+doxycycline group was less, but the difference was not significant.

### 3.2. Body Weight Change

There was no significant difference in average weight among the first five groups. The body weight loss of rats was significant in all groups before the relaparotomy (Figure 2(b)), which may be due to colon resection and its effect on the normal diet of rats. According to the body weight changes between the beginning of the study and before the first operation in groups 4 and 5, it can be seen that preoperative CRT reduced the body weight of rats significantly (Figure 2(b)). The use of MnTE-2-PyP did not have a significant effect on the weight of rats.

### 3.3. MnTE-2-PyP Increased Anastomotic Strength

**Figure 3:**
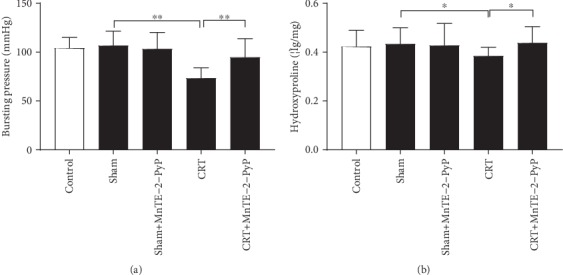
MnTE-2-PyP increased anastomotic strength. (a) Anastomotic bursting pressure of rats in the first five groups. (b) Anastomotic hydroxyproline levels of rats in the first five groups. One-way ANOVA was used to calculate statistical significance. Results are presented as means ± SD. ^∗^*P* < 0.05, ^∗∗^*P* < 0.01.

Bursting pressure (BP) of intestinal anastomosis is important for evaluation of anastomotic healing, and hydroxyproline is a marker of collagen deposition at the anastomosis. In the present study, the two indexes were used to evaluate the anastomotic strength. The average BP of anastomosis and anastomotic hydroxyproline levels in each group is shown in Figure 3. BP and hydroxyproline level decreased because of preoperative CRT; however, MnTE-2-PyP maintained BP and hydroxyproline levels similar to controls.

### 3.4. MnTE-2-PyP Inhibited the Expression of MMPs

**Figure 4:**
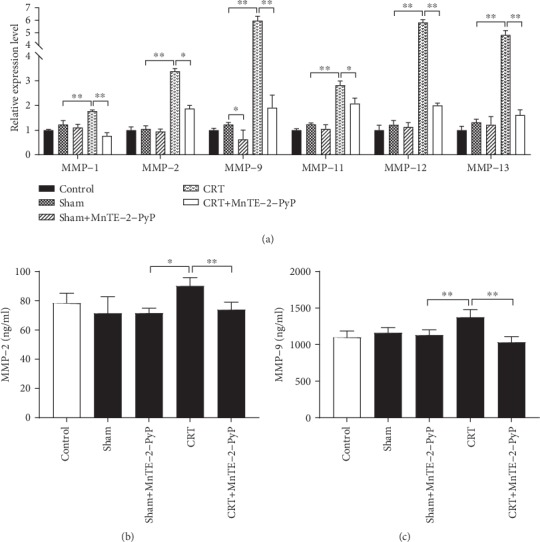
Effect of CRT and MnTE-2-PyP on the MMP expression during anastomotic healing. (a) Indicated MMP mRNA expression was measured by quantitative real-time PCR from colon segments obtained during relaparotomy. (b) MMP-2 levels of experimental groups measured by enzyme-linked immunosorbent assay (ELISA). (c) MMP-9 levels of experimental groups measured by ELISA. (a) Unpaired Student's *t*-test, (b, c) one-way ANOVA. Results are presented as means ± SD. ^∗^*P* < 0.05, ^∗∗^*P* < 0.01.

**Figure 5:**
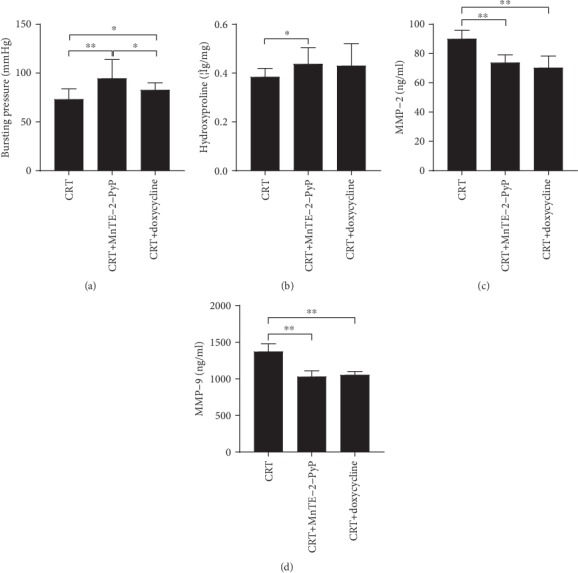
Effect of nonspecific MMP inhibitor doxycycline on anastomosis. (a) Anastomotic bursting pressure of rats in CRT groups (groups 4, 5, and 6). (b) Anastomotic hydroxyproline levels. (c, d) MMP-2 and MMP-9 levels were measured by ELISA after MnTE-2-PyP and doxycycline treatment (groups 4, 5, and 6). One-way ANOVA was used to calculate statistical significance. Results are presented as means ± SD. ^∗^*P* < 0.05, ^∗∗^*P* < 0.01.

RNA was extracted from colon segments in each group, and qRT-PCR was carried out to detect the expression of various MMPs. CRT treatment significantly increased the MMP mRNA levels compared with the sham group, while MnTE-2-PyP decreased the mRNA levels of MMPs (Figure 4(a)). Colon segments were taken from each group, and MMP-2 and MMP-9 levels were measured by ELISA. The expression of MMP-2 and MMP-9 was inhibited by MnTE-2-PyP in group 5 (Figures 4(b) and 4(c)). Doxycycline is a broad-spectrum MMP inhibitor, and its inhibitory effect on MMP-2 and MMP-9 was confirmed (Figures 5(c) and 5(d)). MnTE-2-PyP has similar MMP inhibitory effect to doxycycline. Doxycycline increased only BP but not hydroxyproline levels. Furthermore, its effect on enhancing anastomotic strength was inferior to MnTE-2-PyP (*P* < 0.05, Figure 5(a)).

### 3.5. MnTE-2-PyP Increased Collagen Deposition at the Anastomosis

**Figure 6:**
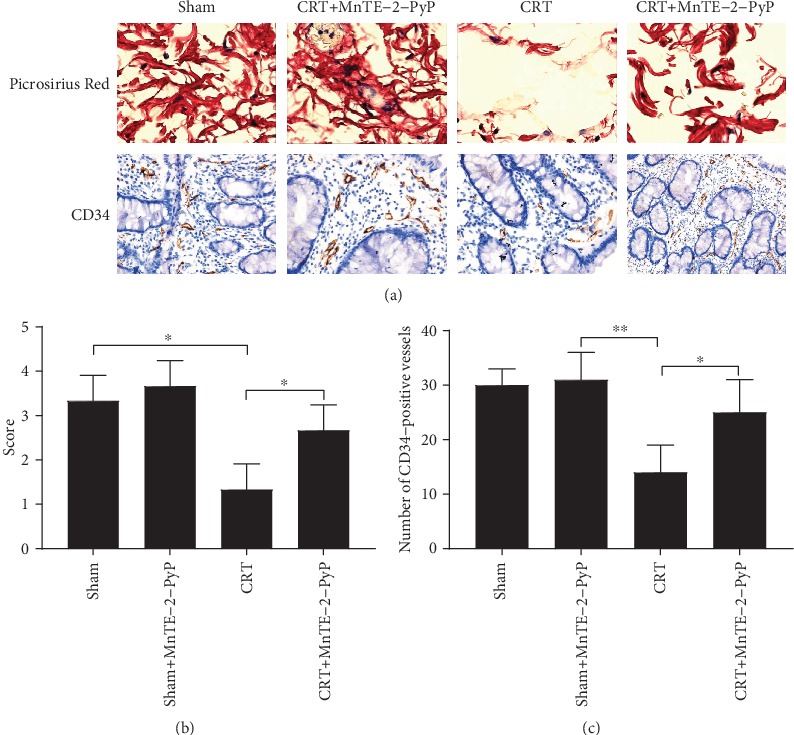
Pathological examination of colon segments of groups 1-5. (a) Representative images of Picrosirius Red staining (upper) and CD34 immunohistochemistry (lower) sections of anastomotic tissues in groups 2-5 (400x). (b) The histological score of anastomotic tissues in each group. (c) Microvessel was measured by the average number of CD34-positive vessels. Microvessel density (MVD) was determined according to the number of stained microvessels per optical field. Unpaired Student's *t*-test was used to calculate statistical significance. Results are presented as means ± SD. ^∗^*P* < 0.05, ^∗∗^*P* < 0.01.

The anastomotic tissues from groups 2-5 were stained with Picrosirius Red. Picrosirius Red was used to observe collagen deposition in anastomotic tissues. As shown in Figure 6(a), collagen fibers in anastomotic tissues were stained red. The median collagen deposition score in the CRT group was significantly lower than that in the sham group, and MnTE-2-PyP significantly increased collagen fiber accumulation (Figure 6(b)).

### 3.6. MnTE-2-PyP Increased Microvessel Density at the Anastomosis

The anastomotic tissues from groups 1-5 were studied immunohistochemically with anti-CD34 antibody. The average number of CD34-positive vessels was used to measure the microvessel, and microvessel density (MVD) was determined by the number of stained microvessels. As shown in Figure 6(a), CD34-positive microvessels were stained brown. CRT significantly reduced the MVD of anastomotic tissue, while application of MnTE-2-PyP increased the MVD (Figure 6(c)).

## 4. Discussion

Surgery is the main method to cure rectal cancer. Preoperative therapy refers to treatment adopted before clinical surgery, including preoperative chemotherapy (CT), preoperative radiotherapy (RT), and preoperative chemoradiotherapy (CRT). With the development of medical technology, preoperative CRT is gradually being used as a standard treatment for LARC. It has been believed that preoperative CRT can reduce the positive rate of surgical resection margin, increase the chance of anal preservation, reduce local recurrence, and improve postoperative survival rate compared with CT or RT alone [24].

Unfortunately, preoperative CRT increases many postoperative complications while improving perioperative efficacy and survival. Many surgeons and scholars believe that CRT increases the incidence of postoperative anastomotic leak (AL) rate [7]; however, some studies suggest that preoperative CRT has no significant effect on AL [25, 26]. The bursting pressure (BP) of intestinal anastomosis is an important index to evaluate the anastomotic healing. In the present study, the CRT group (group 4) had a mean BP that was significantly lower (Figure 3(a)). The data suggested that CRT may have an adverse effect on the healing of intestinal anastomosis in rats.

Some studies have shown that CRT can increase tissue reactive oxygen species (ROS), leading to excessive oxidative stress, tissue destruction, and even toxicity [9, 27, 28]. We deduced that excessive ROS and oxidative stress may be one of the reasons for the increased AL after preoperative CRT. MnTE-2-PyP, a superoxide dismutase (SOD) mimic, can act as a ROS scavenger to protect tissue from radiation damage and accelerate wound healing [13, 15]. It acts as prooxidants by taking advantage of higher hydrogen peroxide levels in cancer cells and promotes cancer cell death when CRT is applied [29]. In our study, application of MnTE-2-PyP in CRT rats of group 5 significantly increased the strength of the anastomosis after colon resection (Figure 3(a)).

MMP is calcium-dependent zinc-containing endopeptidase, which is involved in the degradation of extracellular matrix [30]. The function of MMPs can be influenced by ROS. The inflammatory response at the tumor site can activate neutrophils and macrophages to produce a large amount of ROS. These oxidants activate MMPs through oxidation of prodomain cysteine [31]. As a ROS scavenger, MnTE-2-PyP was used in rats undergoing standard resection and colon anastomotic tissue was taken to observe its effect on anastomotic healing. As shown in Figure 4(a), MnTE-2-PyP significantly reduced the increase of transcription level of MMPs. MMP-2 and MMP-9 are the most widely studied members of the MMP family. According to existing studies, MMP-2 and MMP-9 are overexpressed in colorectal cancer [32], and MMP-9 is a poor prognostic factor in preoperative CRT response of LARC patients [33]. Therefore, we detected the content of MMP-2 and MMP-9 in anastomotic tissues by ELISA; the data were consistent with the results of qRT-PCR (Figures 4(b) and 4(c)). Thus, MnTE-2-PyP may contribute to anastomotic healing through reducing excessive ROS and inhibiting MMPs. Doxycycline, a kind of broad-spectrum tetracycline antibiotic, is a nonselective MMP inhibitor [34]. Doxycycline significantly reduced the level of MMP-2 and MMP-9 in tissues, and the inhibitory effect was similar to that of MnTE-2-PyP (Figures 5(c) and 5(d)). Furthermore, doxycycline increased only BP but not hydroxyproline levels (Figures 5(a) and 5(b)), and its effect on enhancing anastomotic strength was inferior to MnTE-2-PyP (Figure 5(a)); it suggested that MnTE-2-PyP may accelerate anastomotic healing by other mechanisms besides inhibiting MMPs.

Collagen is an essential structural protein for tissue regeneration [35]. Hydroxyproline is a marker of collagen deposition. Some studies showed that RT increases wound hydroxyproline [36], while others found that RT had no systemic effect on wound collagen deposition [37]. In the present study, preoperative CRT significantly decreased hydroxyproline levels. Interestingly, MnTE-2-PyP maintained the hydroxyproline levels similar to controls (Figure 3(b)). In addition, Picrosirius Red was used to observe the deposition of collagen in anastomotic tissues pathologically. As shown in Figures 6(a) and 6(b), CRT reduced collagen while MnTE-2-PyP increased collagen accumulation in anastomotic tissues compared to the sham group. MnTE-2-PyP promoted wound healing, and this effect may be associated with increased collagen deposition. However, we have not yet demonstrated that changes in collagen deposition are associated with changes in MMP levels in anastomotic tissues.

The growth of new capillaries or angiogenesis indicates the feature of wound healing [38]. In wound healing of normal tissues, angiogenesis can bring nutrients, immune cells, and oxygen to the wound, which is very important for wound healing [39]. It has been gradually recognized that oxidative stress plays a positive role in angiogenesis; the main mechanism involves hypoxia-inducible factor/vascular endothelial growth factor (VEGF) signaling [40]. Microvessel density (MVD) has been the most widely used parameter to evaluate angiogenesis in tumor [41]. In many studies, inhibition of MMP-2 and MMP-9 decreased tumor angiogenesis [42, 43]. However, in the present study, MnTE-2-PyP inhibited MMPs of anastomosis (Figures 3 and 4) but increased MVD (Figures 6(a) and 6(c)). The reason for this contradiction may be that ROS act as a double-edged sword in the vasculature. Abnormally high concentrations of ROS can be detrimental for most tissues, whereas low levels of ROS are able to activate signaling pathways that ultimately promote regeneration and growth [44]. In addition, it may be related to the stability of oxidative stress in tissues [45].

In conclusion, the application of CRT before colon resection increased the incidence of postoperative AL in rats, while MnTE-2-PyP enhanced the anastomotic strength. Specifically, MnTE-2-PyP inhibited MMPs and accelerated anastomotic healing by promoting angiogenesis and increasing collagen deposition. Therefore, MnTE-2-PyP is expected to be beneficial in the prevention of postoperative AL after preoperative CRT.

However, there are still weaknesses in our study. For example, we established the colon anastomosis model but not the rectal anastomosis model because of the particularity of rat anatomy. There was no statistical difference in the incidence of AL among the six groups; an increase in the number of rats might make the study more convincing.

## Data Availability

The datasets used and analyzed during the current study are available from the corresponding authors on reasonable request.

## Abbreviations

AL: Anastomotic leak
BP: Bursting pressure
RC: Rectal cancer
CRT: Chemoradiotherapy
CT: Chemotherapy
DNA: Deoxyribonucleic acid
ECM: Extracellular matrix
ELISA: Enzyme-linked immunosorbent assay
HE staining: Hematoxylin-eosin staining
IL: Interleukin
MMP: Matrix metalloproteinase
MnP: Manganese- (Mn-) substituted N-pyridylporphyrin
MVD: Microvessel density
NCCN: National Comprehensive Cancer Network
PBS: Phosphate-buffered saline
qRT-PCR: Quantitative real-time quantitative PCR
RNA: Ribonucleic acid
ROS: Reactive oxygen species
RT: Radiotherapy
SD: Standard deviation
SOD: Superoxide dismutase.
